# CXADR: From an Essential Structural Component to a Vital Signaling Mediator in Spermatogenesis

**DOI:** 10.3390/ijms24021288

**Published:** 2023-01-09

**Authors:** Yang Zhang, Wing-Yee Lui

**Affiliations:** School of Biological Sciences, The University of Hong Kong, Pokfulam, Hong Kong, China

**Keywords:** coxsackievirus and adenovirus receptor, cell junctions, spermatogenesis

## Abstract

Canonical coxsackievirus and adenovirus receptor (CXADR) is a transmembrane component of cell junctions that is crucial for cardiac and testicular functions via its homophilic and heterophilic interaction. CXADR is expressed in both Sertoli cells and germ cells and is localized mainly at the interface between Sertoli-Sertoli cells and Sertoli-germ cells. Knockout of CXADR in mouse Sertoli cells specifically impairs male reproductive functions, including a compromised blood-testis barrier, apoptosis of germ cells, and premature loss of spermatids. Apart from serving as an important component for cell junctions, recent progress has showed the potential roles of CXADR as a signaling mediator in spermatogenesis. This review summarizes current research progress related to the regulation and role of CXADR in spermatogenesis as well as in pathological conditions. We hope this review provides some future directions and a blueprint to promote the further study on the roles of CXADR.

## 1. Introduction

Testes are responsible for the production of male gametes and the sex hormone testosterone. Seminiferous tubules of the testis have some unique features enabling the development and differentiation of male gametes. One of these unique structures is named the blood-testis barrier (BTB), which is one of the tightest blood-tissue barriers in our body and divides the seminiferous epithelium into basal and adluminal compartments, thus creating a distinct microenvironment for germ cell differentiation [1]. Spermatogonia, pre-leptotene, and leptotene spermatocytes reside in the basal compartment, whereas late spermatocytes and spermatids are in the adluminal compartment of the seminiferous epithelium. The BTB is different from other blood-tissue barriers in terms of junction compositions. Apart from having typical tight junctions, the BTB also comprises an array of other junctions including basal ectoplasmic specialization (ES, specialized adherens junctions), desmosomes, and gap junctions [2]. All these junctions are formed between adjacent Sertoli cells near the basal region of the seminiferous epithelium and are coordinated in a way to form a functional barrier. At stage VIII of the seminiferous epithelial cycle, pre-leptotene and leptotene spermatocytes residing in the basal compartment must enter the adluminal compartment for further development [3]. Therefore, cell junctions at the BTB must be restructured in a timely and coordinated manner. At the same stage, there is another cell junction restructuring event that occurs at the apical ES (specialized cell junctions formed between elongate spermatids and Sertoli cells) to allow the timely release of mature spermatids from the seminiferous epithelium at spermiation.

Both BTB and apical ES are made of an array of junction proteins including various transmembrane and peripheral proteins. They coordinate with each other to exert special functions, yet they have different regulatory mechanisms to control their bioavailability and turnover. Although numerous transmembrane proteins are present at the BTB and apical ES, many of them are indispensable in spermatogenesis as a single knockout (KO) of certain transmembrane proteins can result in male infertility, indicating that the function and role exerted by certain transmembrane proteins cannot be replaced by other components. Understanding each component of the BTB and apical ES as well as the way they interact and regulate will uncover the causes of some forms of male fertility and provide a better understanding on spermatogenesis.

Coxsackievirus and adenovirus receptor was initially identified as a common receptor for coxsackievirus and adenovirus [4]. It is expressed in various tissues such as the heart and testis. CXADR is found in both embryonic and adult testis, but the expression declines with age [5,6]. CXADR is expressed by both Sertoli cells and germ cells [5]. The CXADR signal was found to be concentrated at the BTB and apical ES of stage VII-VIII seminiferous tubules [5]. As a validated component of tight junctions, studies have also uncovered that CXADR might function as a regulatory hub for other cell junctions including gap junctions, desmosomes, and adherens junctions. This raised the possibility that CXADR might be a signaling mediator and modulator during the restructuring of the BTB and apical ES at stage VIII of the seminiferous cycle.

This review summarizes current progress about CXADR, mainly focusing on its structure and regulations as well as its functions in various biological process. Based on current progress, we provided some future directions, raised some questions, and made some speculations about the research pertinent to CXADR.

## 2. Structure of the CXADR Protein and Gene

### 2.1. Structure of the CXADR Gene

In humans, the *CXADR* gene encodes the CXADR protein, which is located on chromosome 21, comprising 8 exons (https://www.ncbi.nlm.nih.gov/gene/1525, accessed on 15 December 2022). The promoter region, with a length of 186 bp, is located between −585 to −400 bp upstream of the start codon [7]. Five protein-encoding transcript variants are generated via alternative splicing, which encode for two integral membrane isoforms and three soluble isoforms. Human membranous isoforms, hCXADR1 and hCXADR2, are made up of 365 and 362 amino acids, respectively, and differ only in the last 26 amino acids of their carboxyl-terminus. The identified soluble isoforms are generated by exon skipping. For instance, hCXADR2/7 is formed by alternative splicing between exon 2 and exon 7 [8]. The same rule applies to hCXADR3/7 and hCXADR4/7 [8].

In mice, the ortholog is *Cxadr* (https://www.ncbi.nlm.nih.gov/gene/13052, accessed on 15 December 2022), which is located on chromosome 16. It was claimed that mouse *Cxadr* contains 8 exons [9]; however, 9 exons are annotated in the Ensembl genome browser for the mouse reference genome (GRCm39) [10]. By alternative slicing, the three protein-encoding isoforms are mCXADR1, mCXADR2, and mCXADR3 [9,11].

### 2.2. Structure of the CXADR Protein

CXADR is a highly evolutionarily conserved protein among humans, mice, rats, and bovine. Canonical hCXADR is a transmembrane protein comprising a 19-amino acid (aa) signal peptide, a 218-aa extracellular domain (ECD), a single membrane-spanning segment with a length of 21 aa, and a 107-aa intracellular domain (ICD) [4].

The ECD of CXADR consists of two immunoglobulin-related structural domains (CXADR-D1 and CXADR-D2) [12]. The CXADR-D1 domain spans amino acid residues from 20 to 135. These residues form a β-sandwich fold with the characteristic of Ig variable domains [13]. CXADR exerts its cell adhesion function with adjacent cells through cis-dimerization of CXADR-D1 [14,15,16]. CXADR-D2 contains six β-strands numbered as A (residues 145 to 151), B (residues 156 to 163), C (residues 172 to 178), E (residues 196 to 201), F (residues 206 to 215), and G (residues 220 to 230) [13]. Those β-strands form two β-sheets with one having strands A, B, and F and another having strands C, E and G. Two β-sheets are stabilized by two disulfide bonds, which finally form a β-sheet sandwich motif. Besides, residues 185–192 form a helix named as helix D. Helix D may provide interaction site for CXADR interacting partners [17]. Apart from interacting with junction proteins, ECD of CXADR is also responsible for the interaction of extracellular matrix proteins via homo- and heterodimerization [18].

The ICD of CXADR contains an S-acylation site (cysteine 259 and cysteine 260) for palmitoylation, which is important for the internalization and recycling of the membrane-bounded form of CXADR [19]. Besides, the ICD interact with PDZ-domain-containing proteins via the PDZ binding motif [20]. For instance, cellular scaffolding proteins, such as membrane-associated guanylate kinase with inverted domain structure-1 (MAGI-1) and Ligand-of-Numb protein-X (LNX), bind and interact with CXADR via its PDZ domain [21,22]. Such interaction between CXADR and PDZ-domain-containing proteins are known to regulate the localization of CXADR [23]. Studies have also revealed that CXADR interacts with MAGI-1 via PDZ2 and PDZ4 domains, which can increase or decrease adenovirus transduction, respectively [24]. Apart from interacting with PDZ-domain-containing proteins, the ICD of CXADR also provides multiple regions that are essential for its basolateral sorting [25]. It is apparent that the ICD not only functions as a domain for peripheral protein attachment, but also plays a role in signaling transduction.

## 3. CXADR Knockout and Overexpression Mouse Models

Establishing and investigating CXADR mouse models are of utmost importance to explore the role of CXADR in different developmental processes and to uncover any potential pathogenesis pertinent to CXADR.

Several laboratories have generated numerous CXADR mouse models, including conventional and conditional knockout models, to unravel the physiological roles of CXADR. Two conventional CXADR knockout models with different exons replaced with a neomycin resistance cassette were generated by Asher et al. and Dorner et al. in 2005 [26,27]. The exon 2 excision model demonstrated that CXADR deficiency leads to embryonic lethality. CXADR deficiency mice exhibit severe cardiac defects with regional apoptosis of cardiomyocytes at about 10.5 days post coitum (dpc). All CXADR-deficient mouse fetuses suffered from thoracic hemorrhaging and degeneration of the myocardial wall, leading to death by 11.5 dpc [26], whereas no placenta abnormalities have been observed [26]. Targeted disruption of CXADR via exon 1 excision also causes embryonic lethality at E11.5–E13.5 [27]. Knockout mice exhibit abnormal heart formation and pronounced defects in cardiomyocytes with diminished density of myofibrils, resulting in heart insufficiency [27].

Subsequent studies by Marsman et al. revealed that CXADR is a modifier of cardiac conduction and arrhythmia vulnerability. They found that haplo-insufficient Cxadr (Cxadr^+/−^) mice displayed slowing of ventricular conduction in addition to an earlier onset of ventricular arrhythmias during the early phase of acute myocardial ischemia [28]. Although connexin 43 expression is unaffected, Cxadr^+/−^ mice display increased arrhythmia susceptibility on pharmacological electrical uncoupling and Cxadr^+/−^ myocytes show reduced sodium current magnitude at the intercalated disk [29].

The above three conventional knockout studies unequivocally confirmed that CXADR is crucial for early embryonic cardiac development and is a genetic determinant of arrhythmia susceptibility. However, the roles of CXADR in other organs remain unexplored; embryonic lethality caused by CXADR knockout make it difficult for research to explore the physiological significance of CXADR in other organs. To perform in-depth studies of the role of CXADR in cardiac functions at different embryonic and adult stages, several conditional CXADR knockout mouse models have been generated (Table 1). Research focusing on the role in cardiac development and pathogenesis using these conditional knockout models has been extensively reviewed [30], readers are encouraged to refer there.

Other conditional or cell type-specific CXADR knockout models have been established to investigate the function of CXADR in placenta formation, podocyte function, and male reproduction. Among the tamoxifen-inducible CXADR knockout studies, one study has investigated the reproductive functions upon the administration of tamoxifen [28,39]. Adult male conditional knockout mice (4 weeks old) were injected with tamoxifen once a day for five consecutive days, while prepubertal knockout mice were generated by injecting single dose of tamoxifen at postnatal day 8 [28]. This study found that tamoxifen-induced CXADR-depleted testes display normal sperm parameters like sperm titers and sperm morphology. Histologically, cell junctions at the blood-testis barrier of seminiferous epithelium are unaffected in tamoxifen-induced CXADR-depleted testes. The BTB integrity and function was investigated and no apparent abnormality is observed. CXADR-null sperm can also fertilize normal oocytes [28]. This tamoxifen-inducible CXADR knockout study concluded that CXADR is not essential for male reproductive function.

**Figure 1:**
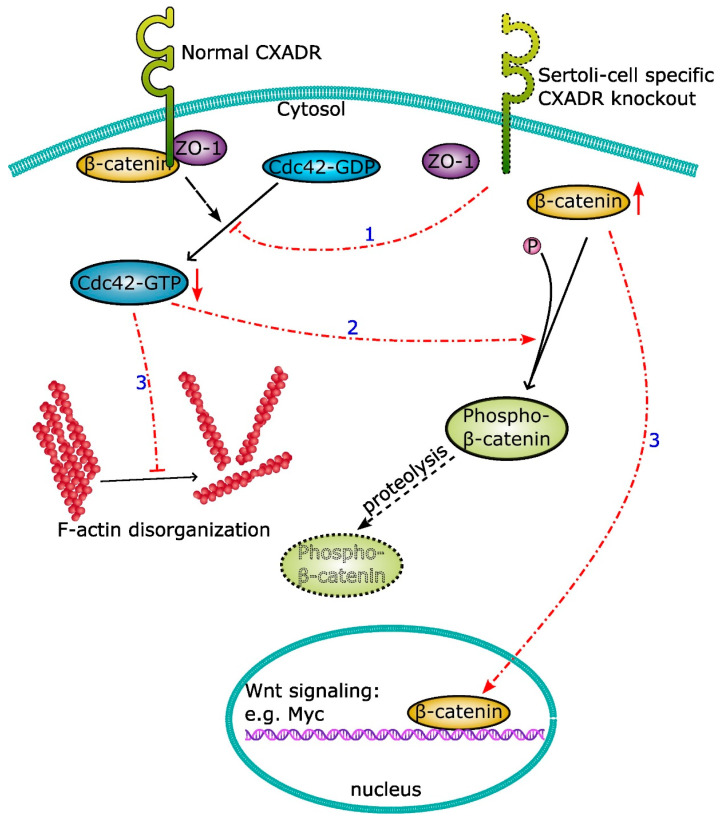
A schematic drawing illustrating the validated regulatory mechanisms of Sertoli cell-specific CXADR-mediated junction dynamics. In normal physiological conditions, Sertoli cell-specific CXADR physically interacts with ZO-1 and β-catenin, which is required for the activation of Cdc42, thus maintaining the organization of F-actin at cell junctions. Sertoli cell-specific CXADR knockout disrupts the distribution of ZO-1 and β-catenin (1). Absence of Sertoli cell-specific CXADR causes a switch of Cdc42 from its active form (Cdc42-GTP) to the inactive form (Cdc42-GDP) (2). Inactivation of Cdc42 results in disruption of the F-actin organization and suppression of β-catenin phosphorylation (3). SC-CXADR knockout also promotes β-catenin translocation from the cytosol to nucleus, hence altering the transcription of its downstream targets such as Myc (3).

Apart from the tamoxifen-inducible CXADR knockout models, two testicular cell type-specific CXADR mouse models named germ cell-specific and Sertoli cell (SC)-specific CXADR knockout have been generated [47]. Two loxP sites flanking exon 3 of the Cxadr gene are inserted to generate floxed mice. Floxed mice were then crossed with stimulated by retinoic acid 8 (Stra8)-Cre and anti-Mullerian hormone (Amh)-Cre mouse strains, respectively, to generate germ cell-specific knockout (GC-CXADR, Cxadr^flox/flox^; Stra8-iCre) and Sertoli cell-specific CXADR knockout (SC-CXADR, Cxadr^flox/flox^; Amh-iCre) mice [47]. GC-CXADR mice display no apparent abnormality in testicular size and tubular morphology compared to age-matched controls [47]. There was no observable difference in terms of fertility in 180-day-old male germ cell-specific CXADR knockout mice compared to controls. However, the fertility of male SC-CXADR mice was significantly impaired [47]. SC-CXADR mice displayed several significant morphological changes including reduced testis-to-body weight ratio and tubular atrophy [47]. Fluorescence-activated cell sorting (FACS) analysis confirmed that there was a significant drop in the number of haploid spermatozoa in the testis and epididymis of male Sertoli cell-specific CXADR knockout mice. The integrity assay revealed that the BTB was compromised with increased barrier permeability. ZO-1 and occludin were downregulated and mis-localized [47]. It is apparent that CXADR in Sertoli cells play an indispensable role in male fertility (Figure 1). Subsequent transcriptomic and proteomic analyses suggested that CXADR in Sertoli cells not only serves as a structural protein at the BTB and apical ES, but also functions as a signaling platform to transduce signaling to regulate other cellular events [47]. For instance, CXADR in Sertoli cell is essential for β-catenin inactivation, cdc42 activation, and inhibition of myc gene transcription [47].

Caruso et al. generated a cardiac-restricted, doxycycline-regulated, CXADR overexpression mouse model [50]. This CXADR overexpression model develops severe cardiomyopathy and mice died at 4 weeks. An obvious disorganization and degeneration of cardiomyocytes have been reported. In this overexpression model, the expression of N-cadherin and β-catenin are altered, resulting in disruption of the adherens junctions [50]. Nuclear translocation of β-catenin and the activation of β-catenin downstream targets like c-myc are also reported [50]. Changes observed in this overexpression model can be rescued by suppressing CXADR expression using doxycycline [50]. Taken collectively, data obtained from the CXADR overexpression mouse model and SC-specific CXADR knockout model unequivocally prove that CXADR is a crucial regulator to modulate β-catenin signaling and adherens junctions.

## 4. Pathogeneses Associated with CXADR Dysregulation

Studies using CXADR knockout and overexpression mouse models have uncovered the physiological roles of CXADR in various organs and shed new insights of potential pathogeneses that may develop in humans with CXADR dysregulation.

Mouse models of myocarditis induced by immunization of cardiac myosin showed a high level of CXADR [51]. Targeted overexpression of CXADR in cardiomyocyte induced cardiomyopathy [50]. In humans, an increase in CXADR expression has also been reported in myocardial diseases such as dilated cardiomyopathy [52,53], and CXADR expression is also observed in infract zones after myocardial infraction [54]. It is apparent that cardiac pathogenesis is related to elevated CXADR expression; however, the mechanisms have not been completely explored. In addition, low CXADR expression levels are found to be associated with arrhythmia vulnerability and cardiac conduction failure. Patients with low levels of CXADR also encounter difficulty in recovery from cardiomyopathy [29,55,56,57]. Cardiac CXADR expression levels seem to be a crucial factor in determining the functionality of cardiomyocytes.

The expression level of CXADR in cardiomyocytes and platelets with virus persistence is highly correlated to the expression level of inflammatory cytokines and the degree of inflammatory dilated cardiomyopathy [58]. CXADR-positive endomyocardial patients express a significantly higher level of tumor necrosis factor α (TNFα) and interleukin 6 (IL-6) in the serum compared with CXADR-negative patients and healthy subjects [58]. This report sheds light on the relationship between inflammation and CXADR.

Apart from cardiac pathogenesis, CXADR may be a potential marker in pertinent to the development and progression of neurodegenerative disease [59,60]. The CXADR expression level was found to be reduced significantly in the human brain at the Braak IV stage (early stage) of late-onset Alzheimer’s disease (AD) [59,61]. A similar reduction of CXADR is observed in triple-transgenic AD mice and a more significant reduction in hippocampi upon systemic inflammation [59,61]. It is believed that CXADR loss might contribute to cognitive deficits in both healthy and disease-primed individuals.

## 5. Functions of CXADR

Aside from being a common receptor for adenovirus and coxsackievirus, numerous in vitro studies have been performed and revealed that CXADR is a multifunctional protein. It interacts with different proteins to trigger and regulate various cellular functions.

### 5.1. CXADR Functions as Structural Component of Tight and Adherens Junctions

In non-polarized cells, CXADR is localized at the interface between cells. Through homotypic interaction, CXADR promotes cell aggregation. Immunofluorescence staining showed that CXADR colocalizes with ZO-1 via its cytoplasmic domain [62]. The expression of CXADR induces re-localization of ZO-1 in non-polarized cells [62]; however, overexpression of CXADR exerts no effect on the expression of ZO-1 in the heart [50]. Based on CXADR knockout studies, CXADR does not appear to be essential in tight and adherens junctions in all epithelium/endothelium. For instance, an intact endothelium and epithelium in some tissues have been observed in various conventional CXADR knockout models [26].

The physiological significance of CXADR as a cell junction component is cell type-dependent. For example, CXADR is expressed by both Sertoli cells and germ cells in the testes [5]; however, apparently, CXADR is not essential for germ cells. Both CXADR-null sperms from Cxadr^flox/+^; Prm1-Cre knockout and GC-CXADR mice can fertilize the oocyte and produce viable litters [31,47,48]. Besides, GC-CXADR male mice display a normal seminiferous epithelium and CXADR-null sperms are able to form functional cell junctions with Sertoli cells at the apical ES so as to hold elongated spermatids with the seminiferous epithelium until spermiation.

In contrast to the germ cell-specific CXADR knockout, it seems clear that CXADR is essential for normal Sertoli cell function [47]. CXADR in Sertoli cells not only serves as an essential building block of tight junctions at the BTB, but also as a protein component of the apical ES to allow elongated spermatids to attach to the seminiferous epithelium prior to spermiation [47]. Inclusion of FTIC-inulin into the adluminal compartment of the seminiferous epithelium in SC-CXADR mice revealed that loss of SC-CXADR causes disruption of the BTB integrity, while a significant decrease in the haploid spermatid population in FACS analysis indicated that loss of SC-CXADR may alter the structure of apical ES and lead to the premature release of spermatids from the seminiferous epithelium and a reduced spermatid number in the epididymis [47]. Morphological changes in the seminiferous epithelium and reproductive abnormality become more apparent and severe with age, resulting in fertility impairment [47,48]. Different testicular cell-specific CXADR knockout models unambiguously proved that CXADR in Sertoli cells, but not in germ cells, is a key structural component at the BTB and apical ES and plays an indispensable role in male fertility.

### 5.2. CXADR Participates in and Regulates the Formation of Other Cell Junctions

CXADR has been found to be an essential tight junction protein in epithelia of several tissues via its interaction with occludin and ZO-1 [62,63]. On top of tight junctions, other studies have revealed that CXADR is also involved in other cell junctions such as gap junctions. CXADR physically interacts with connexin 45, which is a structural component of gap junctions [35]. This interaction requires the PDZ-binding motifs at the carboxyl terminus of both CXADR and connexin 45 [35]. Conditional knockout of CXADR in cardiac cells using α-myosin heavy chain-Cre (α-MHC-Cre) disrupts the localization of connexin 45 in the atrioventricular node [35]. Another cardiac-inducible CXADR knockout mouse model study reported that cardiac CXADR knockout exerts a selective effect on the expression of connexin subsets [36]. Both connexin 45 and connexin 43 are significantly downregulated in CXADR knockout hearts, while connexin40 level remains steady in the CXADR knockout model [36]. connexin 43 and connexin 45 are co-expressed in many tissues, including cardiac cells. They are not only capable of forming homomeric channels, but also work in concert to form heteromeric gap junctions [64]. Carboxyfluorescein dye-coupling assays have revealed that there is potential cross-talk between the tight and gap junctions observed as an increase in dye coupling in CXADR knockout cardiac slices 3 weeks after inducing CXADR knockout [36]. CXADR is required to maintain functional gap junctions at the intercalated disc.

An interplay between CXADR and components of desmosomes has also been reported. Either simultaneous or single knockdown of desmocollin-2 and desmoglein-2 perturbs the localization of CXADR at the interface between cells with no detectable changes in CXADR protein level [65]. Recently, using the proximity-dependent biotin identification (BioID) assay, our lab has identified that desmoplakin and plakoglobin, two essential components of the desmosomes, are the potential interaction partners of CXADR in mouse Sertoli cells [65]. To further evaluate the interactome of CXADR, we generated GST-fused CXADR and performed a GST pulldown assay using human 293T cells; we also successfully pulled down both desmoplakin and plakoglobin. Using two distinct methods, BioID and GST pull down, in two cell lines from different mammalian species, we are able to identify desmoplakin and plakoglobin (unpublished data). These results strongly suggest that CXADR physically interacts with components of desmosomes and is involved in desmosomal function. It is a completely new research area that is worth exploring. In particular, the BTB is formed by coexisting tight junctions, testis-specific adherens junctions, gap junctions, and desmosomes [66]. The possible interplay between CXADR and other junction components suggests the potential role of CXADR as a hub in coordinating the restriction of different junction types at the BTB to accommodate the transit of preleptotene and leptotene spermatocytes from the basal to adluminal compartments at stage VIII for further development.

### 5.3. An Emerging Role of CXADR as a Signaling Mediator

Early studies on CXADR focused mainly on its role as a virus receptor and how it is involved in virus infection. More recently, studies have identified that CXADR is a cell junction component and most subsequent studies have shifted to explore its role in cell junction formation and to unravel its protein interactions. An in-depth investigation of CXADR in pertinent to junction restructuring will help to unfold its emerging role as a signaling mediator.

As a virus receptor, signaling transduction occurs when an adenovirus and coxsackie virus protein binds to CXADR. Upon virus binding, CXADR triggers and activates extracellular signal-regulated kinase ERK1/2 and c-Jun N-terminal protein kinase (JNK) of mitogen-activated protein kinase (MAPK) pathways [49], thus promoting the nuclear translocation of NF-κB [67]. Nuclear NF-κB in turn regulates the transcription of chemokines, including interleukin 8.

The PDZ motif of CXADR binds to and interacts with various cytosolic proteins. Many of these cytosolic proteins, such as β-catenin, are not only peripheral junction components, but also well-studied signalling molecules that regulate various cellular functions apart from junction restructuring. β-catenin is a known interacting partner of CXADR [47,68] and plays a crucial role in Wnt/β-catenin signaling [69]. Altered CXADR expression significantly modulates Wnt/β-catenin signaling. Studies have revealed that elevated CXADR expression triggers the accumulation of β-catenin in the cytosol and the translocation of β-catenin from the cytosol to the nuclei in the heart [50]. Increased CXADR promotes the interaction between β-catenin and the transcription factors of the T cell-factor/lymphoid-enhancer-factor family. Downstream targets of β-catenin, such as c-myc and BMP4, can be significantly regulated by the overexpression of CXADR [50]. In addition, elevated CXADR promotes the phosphorylation of Akt, which in turn phosphorylates GSK-3β. Phosphorylated GSK-3β has limited ability to phosphorylate β-catenin. These results suggested that the phosphorylation status of β-catenin can be regulated by CXADR-mediated Akt-GSK-3β signaling [50]. The phosphatase and tensin homolog (PTEN), which is an upstream regulator of AKT, is also a CXADR-interacting partner [70]. Studies have shown that the expression of PTEN is highly regulated by CXADR. An elevated CXADR level promotes the expression of PTEN while knockdown of CXADR suppresses PTEN expression. Thus, CXADR regulates the AKT signalosome via its precise regulation of the stability and function of PTEN [70].

CXADR signalling also exerts an effect on the stability of epithelial cell junctions. Studies have demonstrated that phosphorylation of CXADR at the carboxyl-terminus signals and mediates the endocytosis of E-cadherin at the site of the cell-cell interface [71]. Studies performed in epithelial cells suggest that CXADR cross-talk with the integrin receptor to facilitate viral entry via CXADR-induced P44/42 MAPK activation. Upon CXADR-mediated activation of P44/42 MAPK, beta1 and beta3 integrins are activated and localized to the site of cell-cell contact [72]. Taken together, CXADR is undoubtedly an emerging signalling molecule that triggers and/or works in concert with different signalling molecules to trigger various downstream effects.

## 6. Regulation of CXADR

CXADR is highly expressed in the heart, brain, and testis during embryonic development. Its expression level drops significantly after birth in certain organs such as the heart [5,16,73]. In the testis, CXADR expression decreases with age [5]. CXADR also exhibits a cyclic expression during the seminiferous epithelial cycle under precise regulatory mechanisms. The precise regulation of CXADR is crucial for BTB and apical ES restructuring. Apart from the testis, CXADR exhibits various functions in different organs. Studies have reported that physiological abnormalities such as cardiac failure, impairment in spermatogenesis, and tumour development are related to altered CXADR levels [47,50,58,63]. It is apparent that proper expression of CXADR in various organs is crucial for normal body function. Studies have uncovered that various regulatory mechanisms mediated by cytokines and microRNAs are involved to regulate the timely and spatial expression of CXADR in various organs, thus exerting normal physiological function.

### 6.1. Ectodomain Shedding and Intramembrane Proteolysis

Protein-protein interaction via the extracellular and intracellular domains of intact CXADR with other proteins are crucial for various CXADR-mediated cellular functions, such as cell adhesion. Apart from function exerted by intact CXADR, cleavage of extracellular and intracellular CXADR produces fragments that may exert downstream regulatory effects and trigger other cellular functions [74]. There is no doubt that cleavage of CXADR at the cell surface provides an effective mechanism to modulate the bioavailability of CXADR. For example, ectodomain shedding of the extracellular domain of CXADR can result in the production of a soluble fragment that functions as a signaling molecule to disrupt cell junctions and limits the susceptibility to virus infection [75]. Various serine proteases, such as neutrophil elastase, cathepsin G, and proteinase 3, are able to induce the cleavage of the ectodomain (ECD) of CXADR [76]. Metalloproteinase like matrix metalloproteinase 3 (MMP-3), but not MMP-1, can trigger CXADR ectodomain shedding in a time- and dose-dependent manner [76]. For instance, neutrophil elastase cleaves the ECD of CXADR within 5 min, which enables a rapid immune response and inhibits viral entry upon infection [76]. It is also known that ADAM10 can cleave CXADR at M224 to L227 in activated glioma cells or developing neurons [74].

Apart from ectodomain shedding, CXADR also undergoes regulated intramembrane proteolysis by γ-secretase [24,74] and β-site amyloid precursor protein-cleaving enzyme (BACE1) [77]. Upon γ-secretase-mediated proteolysis, the intracellular domain (ICD) of CXADR is released and translocated to the nucleus [74], thus potentially exerting other downstream regulation in the nucleus.

Both ectodomain shedding and intramembrane proteolysis of CXADR not only provide an effective mechanism to control its bioavailability at the cell surface, but also, CXADR partial fragments may function as biomolecules to transduce signals to regulate other cellular activities.

### 6.2. Cytokines

Inflammatory cytokines, including tumour necrosis factor α (TNFα) and transforming growth factor β (TGF-β), have been identified as key regulators of cell junction restructuring. Numerous studies have been performed to unfold the mechanisms of how these biomolecules regulate the expression and localization of various junction components in different epithelial cells. Surprisingly, investigations on CXADR regulation are very limited.

In chronic lung inflammation models, TNFα exerts no effect on the expression level of CXADR but promotes the phosphorylation of CXADR via the phosphoinositide 3-kinase (PI3K)-PKCδ pathway [78]. Primary Sertoli cells have been found to be responsive to TNFα stimulation. A significant reduction in the CAXDR protein level was observed in primary Sertoli cells upon TNFα treatment (20 ng/mL) [5].

TNF-α itself exerts a slight downregulatory effect on the CXADR protein level in mouse germ cells [79]. This downregulation can be significantly enhanced if cotreating with interferon-γ (IFN-γ, 5 ng/mL), while IFN-γ alone exerts no detectable effect on the expression level of CXADR. Combined treatment of TNFα and IFN-γ disrupted the localization of CXADR at the interface between cells. It has been found that ubiquitin-proteasome pathway and nuclear factor κB (NF-κB) are engaged in TNFα+IFN-γ-induced CXADR protein degradation. Besides, TNFα+IFN-γ also suppresses the basal transcription of CXADR, leading to the reduction the mRNA level of CXADR in germ cells. Apart from testicular cell models, similar negative regulatory effects of TNFα+IFN-γ on CXADR are reported in in hippocampal neurons as well as in adult murine neural progenitor cells [59].

### 6.3. Components of Cell Junctions

As a component of cell junction, CXADR interacts with many other components of these cell junctions. Due to the intimate interaction between various cell junction proteins, it is not surprising that junction proteins localized at the site of cell-cell contact can regulate one another, so as to achieve timely and temporal regulation on junction restructuring.

Membrane-associated guanylate kinase with inverted orientation protein-1 (MAGI-1) is a cytosolic protein that is present at tight junction [80]. CXADR directly interacts with MAGI-1 via the PDZ-1 and PDZ-3 domains of MAGI-1 [81]. MAGI-1 regulates the level of CXADR both in nonpolarized cells and polarized epithelia [23,81]. Binding of CXADR with the PDZ-3 domain of MAGI suppresses apical CXADR protein expression and adenovirus infection, while expression of the MAGI-1 PDZ1 domain can rescue CXADR from MAGI-1-mediated suppression and facilitate adenovirus infection [81]. Another study found that the PDZ-2 domain of MAGI-1 is important to regulate CXADR cleavage. Disruption of the binding of CXADR to the PDZ-2 domain of MAGI-1 induced cleavage of the cytoplasmic domain of CXADR [24]. Binding of CXADR with MAGI-1 via different PDZ domains resulted in differential regulation of CXADR bioavailability.

Apart from MAGI-1, simultaneous knockdown of plakophilin-2 and connexin 43 caused a reduction in the CXADR protein level in primary Sertoli cells [82]. A significant decline in the level of biotinylated cell surface-bound CXADR has been observed upon knockdown of plakophilin-2 and connexin 43 [82], whereas there was no detectable change on CXADR expression and localization in single knockdown of either plakophilin-2 or connexin 43. Another study has reported that single or simultaneous knockdown of both desmoglein-2 and desmocollin-2 disrupts the localization of CXADR and promotes the internalization of CXADR without affecting the total CXADR protein level [65]. These studies not only uncovered that desmoglein-2, desmocollin-2, and plakophilin-2 can regulate the CXADR level, but only shed a new insight on the potential functional relationship/interplay between desmosomes and CXADR-based tight junctions.

### 6.4. MicroRNAs and E3 Ligase

Based on a microRNA array-based screening, miR-466 has been identified as host cell microRNA induced by coxsackie B viruses [83]. Overexpression of rat rno-miR-466 and human has-miR-466 suppressed the protein expression of CXADR [83]; however, the detailed mechanisms underlying the regulation of CXADR by miR-466 remain to be elucidated.

Ubiquitination-mediated protein degradation is a post-translational regulatory approach to effectively regulate the bioavailability of proteins. Ligand-of-Numb protein X1 (LNX1) is an E3 ubiquitin ligase comprising a RING finger domain and four PDZ domains. LNX1 binds to and co-localizes with CXADR in mammalian cells. Further studies have confirmed that LNX1 is the E3 ligase targeting CXADR. Suppression of ubiquitination and degradation of CXADR has been reported in LNX-1 deficient cardiomyocytes [84].

## 7. Future Directions

In the testis, CXADR is not only a putative tight junction protein at the BTB, but also a signaling molecule that regulates cdc42/β-catenin signaling [47]. Numerous signaling pathways that are known to be crucial for spermatogenesis are altered in our SC-CXADR knockout model [47]. Apart from the well-studied TNF and TGF-β signaling pathways in pertinent to the BTB restructuring, our integrated omics analyses have identified that SC-CXADR KO alters Toll-like receptor signaling and the PI3K-Akt pathway [47,79]. Previous studies have revealed that the PI3K-Akt signaling pathway is involved in regulation of the apical ES [85]. It is of interest to know whether SC-CXADR is the upstream signaling mediator to regulate the apical ES via the PI3K-Akt pathway. GO enrichment analyses of both transcriptomics and proteomics have shown that focal adhesion is the highly enriched biological process in SC-CXADR KO model. The structure of the apical ES and the focal adhesion resemble each other in some ways. For example, both have a highly dynamic, actin-mediated structure. Integrins and extracellular matrix proteins are the major structural components found in these two structures. Apart from being a component of the apical ES, what role will SC-CXADR be involved in in regulating the apical ES? Will SC-CXADR interact with integrins expressed in germ cells at the apical ES to exert similar functions to control “focal” adhesion dynamics with migrating spermatids? Apparently, attention should be paid to uncover these novel functions of SC-CXADR in spermatogenesis.

Figuring out the molecular configurations of these CXADR-mediated junctions might provide valuable clues for developing innovative contraceptive drugs or devices. Recent progress demonstrated that cryogenic electron microscopy (cryo-EM) is a magnificent tool for the study of protein complexes [86]. Employing this technique might greatly facilitate the study of structures at the molecular level in the testis. In addition, dissecting the potential interplay between CXADR with desmosomes is another area that deserves attention. Unraveling the cross-talk mechanism between tight junctions and desmosomes via CXADR definitely helps to develop a complete understanding of restructuring event at the BTB at stage VIII of the seminiferous cycle [65]. It is believed that this piece of new information will also shed a new insight to researchers in the field of cell biology.

The difference in the expression pattern of CXADR between embryonic and adult tissues, as well as between pathological and normal tissues, suggests that precise regulation of CXADR is required for normal physiological functions. However, limited studies have been performed so far. Comparing a high level of CXADR in embryonic and inflammatory tissues, as well as during the restructuring of the testicular cell junction, with the relative low expression of CXADR in most adult tissues, suggests that CXADR is a key signaling mediator in initiating tissue repair [59,67]. In addition, it has been reported that CXADR expression alters during tumor development; however, the alteration of CXADR expression seems to be tumor specific. What are the exact roles of CXADR in pathogenesis and pathophysiology? This question should be investigated in detail for the potential clinical applications.

## Figures and Tables

**Table 1:** Phenotypes of current generated CXADR knockout mouse models.

Model	Exon	Time	Phenotypes	Reference
Cxadr^−/−^	2	E0 *	Embryonal lethal between E11.5 and E13.5 Thinner myocardial wall Cardiomyocytes degenerating Cardiomyocytes death	[26]
Cxadr^+/−^	2	E0	Normal	[26]
Cxadr^+/−^	1	E0	Normal	[27]
Cxadr^−/−^	1	E0	Embryonal lethal between E11.5 and E13.5 Reduced density of myofibrils Disorganized orientation and bundling of myofibrils Formation of pericardial edema	[27]
Cxadr^−/−^	2	E0	Embryonal lethal between E11.5 and E12.5, or die shortly after birth Regional over-proliferation of cardiomyocytes Hyperplasia of the left ventricle Abnormal junction between sinus venosus	[31]
Cxadr^flox/flox^; Protamine-Cre	2		Cxadr-null sperm is still fertile	[31,32]
Cxadr^flox/flox^; TNT-Cre	2	E9.5	Embryonal lethality	[31,33]
Cxadr^flox/flox^; α-MHC-Cre	2	E11.5	Viable	[31,34]
Cxadr^flox/flox^; α-MHC-cre	1		Blockage of atrioventricular conduction in the adult heart Prolonged atrioventricular conduction in the embryonic heart Loss of connexin 45 Decreased β-catenin and ZO-1 amount and localization Cardiomyopathy	[34,35]
Cxadr^−/−^	2		Embryonal lethal between E11.5 and E12.5 Hemorrhage Pericardial effusion	[35]
Cxadr^flox/flox^; α-MHC-cre, Tamoxifen-Inducible	1	P2 months	Impaired electrical conductance from the atrium to ventricle Reduced expression of ZO-1 Reduced expression of connexin 45 Altered localization of connexin 43	[36,37]
Cxadr^flox/flox^; α-MHC-cre, Tamoxifen-Inducible	1	P2 months	Prevent signs of inflammatory cardiomyopathy after CVB3	[37,38]
Cxadr^flox/flox^; Cre-ER^TM^	2	P3 weeks	Dilated intestinal tract Atrophy of the exocrine pancreas Abnormal thymopoiesis	[39,40]
Cxadr^flox/flox^; Cre-ER^TM^	2	E12.5	Lethal Subcutaneous edema Hemorrhage Embryonic lethality Dilated subcutaneous lymphatic vessels Abnormal structure with gaps and holes presents at lymphatic endothelial cell-cell junctions Erythrocyte leakage	[39,41]
Cxadr^flox/flox^; Cre-ER^TM^	2	E13.5	Viable	[39,41]
Cxadr^+/−^	1		Slower ventricular conduction Increased arrhythmia susceptibility Reduced sodium current magnitude	[29]
Cxadr^flox/flox^; Cre-ER^TM^	2	P4–6 weeks	Intact blood-testis barrier Uncompromised fertility No detectable phenotype up to the age of 8 months	[28,39]
Cxadr^flox/flox^; Cre-ER^TM^	2	P8	Intact blood-testis barrier uncompromised fertility	[28,39]
Cxadr^flox/flox^; hNphs2-Cre	1	E14.0	Normal podocyte development Normal stress response	[42,43]
Cxadr^flox/flox^; Tnnt2-Cre	1	E7.5	Embryonic lethality by E12.5 Thinner placentas Decreased labyrinth depth	[44,45]
Cxadr^C210A/C210A^-ENU	N.A.		Thinness of the labyrinth	[44]
Cxadr^flox/flox^; Myh-6-Cre	1		Viable No obvious labyrinth defects	[34,44]
Cxadr^flox/flox^; Sox2-Cre	1	E14.5	Lethal between E11.5 and E12.5 Altered interheamal membrane architecture Reduced IHM branching Flatter placentas	[44,46]
Cxadr^flox/flox^; Stra8-iCre	3	P8	No observable changes in reproductive functions;	[47,48]
Cxadr^flox/flox^; Amh-Cre	3	E14.5	Reduced fertility with age Increased germ cell apoptosis Premature loss of elongated spermatids Compromised BTB function and apical ES structure Dysregulation of occludin and ZO-1 Altered β-catenin/Cdc42 signaling	[47,49]

* E: embryonic day; N.A.: not available; P: post-natal.

## Data Availability

No new data were created or analyzed in this study. Data sharing is not applicable to this article.
